# Construct Validity of a Questionnaire on Eating and Physical Activity Habits for Adolescents in Mexico City

**DOI:** 10.3390/healthcare11162314

**Published:** 2023-08-16

**Authors:** Araceli Martínez Coronado, Irina Lazarevich, Rey Gutiérrez Tolentino, Miguel Ángel Mejía Arias, Gerardo Leija Alva, Claudia Cecilia Radilla Vázquez

**Affiliations:** 1 Division of Biological and Health Sciences, Universidad Autónoma Metropolitana-Xochimilco, Mexico City 04960, Mexico; 2 Fundación Aprende con Reyhan A.C., Mexico City 04640, Mexico; 3 Interdisciplinary Center for Health Sciences, Santo Tomás Unit, National Polytechnic Institute, Mexico City 11340, Mexico

**Keywords:** validation, eating, physical activity, habits, adolescents

## Abstract

The assessment of eating and physical activity habits is an important step in promoting healthy behaviors among the adolescent population and is key in the prevention and management of chronic non-communicable diseases, such as obesity, diabetes, and cardiovascular disease. For this purpose, reliable and valid measuring instruments are essential. In this context, the aim of this article is to present the validation of a self-report questionnaire on eating and physical activity habits among adolescents in Mexico City. In order to validate the questionnaire, a cross-sectional study was conducted with a sample of 2710 adolescents between 11 and 12 years of age, the piloting of the questionnaire was carried out in September 2022 with a focus group, and the programming of the anthropometric measurements was established with the Federal Educational Authority of CDMX, as well as the application of the questionnaire to 33 schools, with these activities being scheduled from 7 November 2022 to 3 February 2023 and having an application duration of 15–25 min for each of the groups to which it was applied; the questionnaire that was applied consists of 31 questions that refer to the frequencies, quantity, or performance of behaviors related to the frequency and type of food, type of physical activity and behaviors related to the act of eating referring to the place where it is carried out (home or away from home) and with whom it is carried out (alone or in company), and about the individual’s lifestyle. Subsequently, the reliability of the instrument was evaluated using Cronbach’s alpha coefficient, and an exploratory factor analysis was conducted to determine the structure of the questionnaire. The results obtained showed that the questionnaire was adequately reliable (α = 0.778) with an eight-factor structure: four questions on mealtime frequencies, four questions on physical activity and lifestyles, six questions on the consumption of high-calorie foods, four questions on company and food consumption, four questions on the consumption of vegetables and fruits, four questions on the place of food consumption, two questions on the consumption of alcoholic beverages, and three questions on the consumption of sugary drinks, plain water, and milk. In conclusion, the self-report questionnaire on eating and physical activity habits among adolescents in Mexico City is reliable, has adequate internal consistency, and can therefore be used as a useful tool for the evaluation of eating and physical activity habits in this population.

## 1. Introduction

Obesity has become a worldwide public health problem, especially among adolescents, with its prevalence projected to be more pronounced in 2035, rising from 10 to 20% in males and from 8 to 18% in females compared to 2020 [1]. On the other hand, the National Health and Nutrition Survey 2021 indicates that the prevalence of being overweight and obese in the population aged 12 to 19 years was 42.9% (24.7% overweight and 18.2% obese), with the percent of individuals who are overweight being higher for women (26.4%) compared to men (23.0%); however, for obesity, the percentage is higher in men (21.5%) compared to women (15%); therefore, the problem of obesity in adolescence is related to habits and lifestyles such as the type and frequency of food and drinks consumed, as well as the frequency, duration, and intensity of physical activity [2].

With the advent of the SARS-CoV-2 pandemic, there were major lifestyle changes aimed at reducing viral transmission. Adolescents were subjected to prolonged periods at home due to school closure, significantly altering family dynamics and school and work routines, as well as the time dedicated to physical activity and screen time; these changes were associated with greater sedentariness, increased body weight, and altered sleep schedules and sleep quality, and, likewise, economic stability was affected, access to recommended foods was reduced, and the intake of ultra-processed, non-perishable foods with higher caloric content and high in saturated fats and refined sugars increased [3,4,5].

According to Mexican Official Standard 043 (NOM-043) [6], a healthy diet is one that meets the specific needs of the different stages of life, so as to ensure nutrition and disease prevention. However, eating habits are a set of customs that condition the way in which people select, prepare and consume food, and although eating habits are acquired during childhood and the family plays a fundamental role in shaping eating patterns, upon reaching adolescence, the role of the family loses relevance, and groups of friends and social recommendations become key conditioning factors for adolescent nutrition, and, in addition to this, many educational institutions offer and sell foods with high caloric content and low nutritional value that, in turn, replace the consumption of vegetables and fruits and contribute to the intake of non-recommended foods [7].

Furthermore, low levels of physical activity are also related to being overweight and obese in adolescence [8], which is why the World Health Organization (WHO) recommends that children and adolescents should engage in at least 60 min of moderate to vigorous physical activity, mainly aerobic exercise, and limit time spent carrying out leisure activities or screen time. Therefore, by not having healthy eating habits and opting for a sedentary lifestyle, the health outlook could worsen at this stage and beyond [9,10,11].

In this sense, the adoption of healthy eating habits along with regular physical activity is essential to prevent obesity in adolescence, and 3 years after the pandemic and 1 year after daily activities were restored, it is important to evaluate adolescents at this time in relation to eating habits and physical activity [2]. In the last year, it has been recommended that governments, schools, health professionals, and parents be aware of the serious situation and take more effective actions immediately to minimize the negative impact of the COVID-19 pandemic [12]. Considering that eating and physical activity habits are modifiable behaviors that influence the prevention of chronic non-communicable diseases, their assessment is important for designing strategies to encourage health [13].

To measure adolescents’ eating and physical activity habits, self-report questionnaires have been developed and validated in different contexts and populations. These questionnaires have been developed in Spanish-speaking countries, such as that of Jiménez et al. [14] called the questionnaire to study healthy habits among adolescents aged 12–14 years, which was administered in Murcia, Spain. In the same country, Pérez de Eulate et al. [15] developed a survey on eating habits in Basque adolescents.

However, in Mexico, few instruments for food intake and physical activity in the adolescent population have been validated. In 2016, Flores and Macedo [16] conducted a study whereby they validated an instrument named *Self-Completed Eating Habits for Adolescents in Jalisco Questionnaire*. This instrument showed adequate reliability due to the fact that the factor weights were significant in their respective components. Internal consistency was also reported as good for most sections. Based on this questionnaire, the need to adapt and validate the instrument for adolescents in Mexico City was identified due to the fact that in a study conducted by Barriguete et al. [17], in which eating habits, physical activity, and lifestyles were evaluated in school adolescents from Mexico City and the State of Michoacán, it was found that adolescents from Michoacán had better eating and physical activity habits than adolescents from Mexico City, suggesting that habits are different in the different states of the country.

Due to the above and given the need to have validated and reliable tools to measure the eating habits and physical activity of this population group, the objective of this study was the construct validity of a questionnaire on eating and physical activity habits by administering it among adolescents in Mexico City (CDMX) during the 2022–2023 school year, as part of a strategy to improve the diet and physical activity of these age groups that were affected during the COVID-19 pandemic.

## 2. Methods

### 2.1. Study Design

The study consisted of two stages, with a duration of the 2022–2023 school year from August 2022 to June 2023. The first included the review process of the self-report questionnaire on eating habits validated by Flores and Macedo in 2016 [16]. Derived from this, a pilot test was carried out in September 2022 with a focus group, made up of 5 female adolescents and 5 male adolescents between the ages of 11 and 12, randomly chosen from the same school community in which it was applied. Subsequently, there was the self-report questionnaire on eating and physical activity habits for adolescents in Mexico City, with the objective of knowing their opinion in relation to the questionnaire and identifying aspects to take into account for the modification of the questionnaire. The data collection was carried out through the application of the questionnaire integrated by 31 questions where the following variables were inquired: frequencies of mealtimes; physical activity and lifestyles; consumption of high-calorie foods; company and food consumption; Consumption of vegetables and fruits; place of consumption of food; consumption of alcoholic beverages; and consumption of sugary drinks, plain water, and milk. With the observations issued by the focus group, some modifications were made, such as a change in the wording of some questions to improve their understanding and the incorporation of portions in food so that adolescents could identify their consumption more easily.

After the piloting of the questionnaire with the focus group carried out in September 2022, the programming of the anthropometric measurements and the application of the questionnaire for the 33 schools were established with the Federal Educational Authority of CDMX. These activities are scheduled from 7 November 2022 to 3 February 2023.

The questionnaire to evaluate the eating and physical activity habits of adolescents was applied to a sample of 2710 adolescents from CDMX. The duration of the application of the questionnaire was 15–25 min for each of the groups to which it was applied.

The same day, prior to the application of the questionnaire to assess eating and physical activity habits, anthropometric measurements were also taken with the use of an Inbody-270 body composition analyzer (South Korea) and by using the Who Anthro Plus^®^ program (Geneva, Switzerland) to estimate weight status with the body mass index (BMI) percentiles proposed by the WHO [18]. Anthropometric measurements were taken at the same time of day because the schedule on the days that corresponded to each of the 33 schools was from 8 to 9 in the morning. The adolescents were asked to consume 1 L of water two hours before the measurement of body composition and fast for three to four hours.

The second stage consisted of validating the reliability of the questionnaire, as well as its internal consistency.

In this study, the focus of selection was aggregated units (33 schools in CDMX), in which there were 2710 students with a mean age of 11 years and 7 months. Schools in the following municipalities were included: Álvaro Obregón, Azcapotzalco, Coyoacán, Cuauhtémoc, Gustavo A. Madero, Iztacalco, Iztapalapa, Miguel Hidalgo, Tláhuac, Tlalpan, Venustiano Carranza, and Xochimilco. For the intervention and control of populations, similar characteristics were considered in socioeconomic and race due to the fact that, in Mexico City, the combination of indigenous and mestizos predominates.

Within the inclusion criteria, the full-time schools of CDMX were considered, including adolescents with an age range of 11 to 12 years, who delivered their informed consent letter signed by their parents and who gave their assent to participate. Students with a clinical diagnosis of diabetes, hypertension, metabolic syndrome, and other chronic diseases were considered as the exclusion criteria, as well as students who were pregnant or lactating at the time of the intervention and adolescents who were not given authorization or did not want to participate. As the elimination criterion, students who refused to participate in any component of the study were considered.

### 2.2. Sample Size

To establish the size of the sample, a base of 119 schools in 16 municipalities of CDMX was used, of which 46 schools met the characteristics required for the study in the 2022–2023 school year (data provided by the Federal Education Authority of CDMX).

The selection was based on simple random sampling with a finite population, and the formula of Murray and Larry [19] was used for the calculation:

$$n=\frac{Z_{\alpha }^{2}\cdot N\cdot p\cdot q}{i^{2}(N-1)+Z_{\alpha }^{2}\cdot p\cdot q}$$

where:

n: sample size.
N: population size; a value of 46 is used (full-time schools).
Z: value corresponding to the approximately normal distribution, Zα = 1.62; α = 0.10.
p: expected prevalence of the parameter to be evaluated, if unknown (p = 0.5), which increases the sample size.
q: 1 − p (if p = 50%, then q = 50%).
i: error assumed (10%).
  $$n=\frac{{\left(1.62\right)}^{2}\left(46\right)\left(0.5\right)\left(0.5\right)}{0.01\left(46-1\right)+\left(1.62\right)\left(0.5\right)\left(0.5\right)}=\frac{30.1806}{0.85555}=35.2\approx 33$$

All adolescents participated voluntarily, with informed consent from their mothers, fathers, and/or guardians and with verbal consent prior to participation. This study was reviewed and approved by the Research Ethics Committee of the Biological and Health Sciences Division of the Universidad Autónoma Metropolitana-Xochimilco. The Divisional Council of Biological and Health Sciences of the Universidad Autónoma Metropolitana-Xochimilco, in session 7/21, held on 6 May 2021 through Agreement 7/21.5.4 of the official letter DCBS.CD.157.21 issued the resolution to approve the research project.

### 2.3. Description of the Questionnaire

The version of the Flores and Macedo 2016 [16] self-report eating habits questionnaire was modified for adolescents in Jalisco (Table 1).

The questionnaire is made up of 31 questions divided into four sections, of which 27 questions from the first three sections assess eating habits and 4 questions from the last section assess physical activity habits. The first section is made up of six questions related to the frequency and quantity of food consumption recommended for daily intake. The second section consists of nine questions regarding the consumption of foods that are not recommended for daily intake. The third section contains 12 questions with information regarding frequency, places of food consumption at mealtimes, and company when consuming food at mealtimes. The fourth section of the questionnaire includes four questions on physical activity, which is considered important to evaluate together with the questions on eating habits because they are considered to be significantly related [16].

For the evaluation of eating and physical activity habits, a score of 0 to 3 points was established for single-question items and 0 to 1.5 points for items containing two or more questions. Thus, for section one, the maximum score was 12 points, for section two, it was 21 points, and for section three, it was 18 points, with a maximum of 51 points to evaluate eating habits. For section four, which evaluates physical activity habits, a maximum of 12 points were given.

For the interpretation of eating and physical activity habits, these habits must be classified according to the score achieved by the adolescent compared to the maximum possible score, as shown in Table 2.

### 2.4. Collection of Anthropometric Measurements and Sociodemographic Data

For this study, anthropometric measurements (height and weight) were used, which were previously standardized and taken by a nutritionist and eight interns of the bachelor’s degree in human nutrition program of Universidad Autónoma Metropolitana-Xochimilco.

The participants were measured in the study classrooms and called by roll number so that the measurement was personal and private.

Prior to the intervention, the adolescents were sent an information sheet in which they were asked to consume 1 L of water two hours before the measurement of body composition and fast for three to four hours.

For the weight measurement, the study subjects were asked their date of birth to be recorded on the InBody 270 equipment. Their weight was taken with the subject in an upright and relaxed position, facing the equipment, with their eyes fixed on a horizontal plane, the palms of their hands holding the upper electrodes, and their feet touching the lower electrodes of the equipment and without moving until the equipment indicated to open the arms to take the body composition. Their weight was taken without shoes or socks, with light clothing, and without objects in their hands or pockets such as keys, coins, toys, or any other object that could alter the measurement.

Height was measured to the nearest millimeter using a SECA 213 (Hamburg, Germany) stadiometer with an integrated leveler [20]; the subject was placed standing, without shoes, and with their heels, calves, buttocks, and back leaning fully against the wall so that the midline of the body matched the midline of the stadiometer. The subject’s chin was taken with the left hand to control the head and direct it towards the Frankfort plane, the movable part of the stadiometer was slid with the right hand until it was placed on the crown of the head, and the measurement was recorded.

BMI [weight (kg)/height (m^2^)] was calculated using the weight and height measured, by age and sex. Adolescent weight status diagnoses were estimated with the WHO Antro Plus program with the height-for-age Z-score and BMI-for-age Z-score of the WHO 2007 reference standard [21].

### 2.5. Administration of the Questionnaire

The questionnaire was administered by a team of health professionals, a nutritionist, and eight previously trained interns from the bachelor’s degree in human nutrition program, who went to the 33 schools according to a role agreed with the Federal Education Authority of Mexico City during the 2022–2023 school year. The team of health professionals who administered the questionnaire to the adolescents provided instructions and remained in the classroom during the time the questionnaire was administered to resolve any doubts and verify that the adolescents answered all the questions. The duration of the application of the questionnaire was 15–25 min for each of the groups of the 33 schools to which the questionnaire was applied according to the schedule.

### 2.6. Validation of the Questionnaire

The procedure to ascertain the reliability of the instrument was carried out using Cronbach’s alpha coefficient. Adequate reliability was considered at values from 0.7 to 0.9 [22]; values below 0.7 indicated low reliability, and those above 0.90 indicated redundancy or duplication. In this study, a reliability of 0.778 was obtained, which is considered adequate.

For the exploratory factor analysis, based on the correlation matrix, with varimax rotation, a minimum weight of 0.3 was established as acceptable. The data were analyzed using the SPSS version 23 program.

## 3. Results

Data from 2710 adolescents were analyzed. Table 3 shows that 47.23% were male and 52.76% were female, with a mean age of 11 years 7 months.

The socioeconomic level was evaluated using the NSE questionnaire [23]; the highest percentage was 39.37%, which represents the typical average socioeconomic level.

With respect to the anthropometric data, it was observed that according to the height/age Z-score diagnosis, 84.7% showed adequate height for their age.

Regarding the BMI-for-age Z-score diagnosis, only 45.7% have an adequate weight; this is an indicator that should be addressed since when adding together those who are overweight and obese, 47.7% already have an indicator of a health risk problem.

In addition, these anthropometric results comparing males and females in general present very low differences in terms of height, basal weight, and BMI; however, when comparing the Z-score diagnosis of height and age, it can be observed that females present a risk of short stature 8.3% higher than males, and, on the other hand, the comparison of the Z-score of the BMI/E shows that males have a 8.4% higher risk of obesity compared to females, while the latter present a 3.9% higher risk of being overweight than males.

In Table 4, we can see the percentage of eating habits and the classification of nutritional status. It is noteworthy that both in male adolescents and in female adolescents, the highest percentage leaned towards partially inadequate eating habits; both in adolescents who are underweight and those who are obese, the percentage ranges from 75% to 84%. This is another indicator that must be addressed and generate campaigns to improve this behavior. 

As shown in Table 5, the highest percentage of physical activity habits is concentrated with inadequate physical activity, and from female adolescents with low weight to those with obesity, around 50% fall into this category. Therefore, it would be important to generate more strategies for this situation to change.

The reason why the percentages of physical activity were low can be attributed to the fact that since 2006, various surveys in Mexico have been reporting that the percentages of “inactivity”, “without activity”, or “decrease in activity” have increased in this population, and if we add that during the last 3 years, derived from the COVID-19 pandemic, confinement and inactivity predominated, the consequences of this are reflected in the results obtained [24]. In a study published in 2021 in Mexico with a population of children between 8 and 13 years of age, it was also found that less than a fifth of the participants complied with the WHO physical activity recommendation, which means that the problem starts in childhood [25].

Finally, to find out if the dimensions of physical activity and the consumption of fruits and vegetables were related, a Spearman correlation was carried out, and the correlation coefficients and the statistical significance (*p* < 0.05) can be seen in Table 6.

The results obtained in the test show that, if there is a relationship between the performance of physical activity and the consumption of fruits and vegetables, the correlations that go from 0.132 to 0.150 show low effect sizes, which indicates that the magnitude of this relationship is present and relevant; however, actions should be taken to increase it.

### Results of the Validation of the Eating Habits and Physical Activity Instrument for Adolescents in Mexico City

Based on the questionnaires answered by the adolescents, the reliability analysis of the instrument was carried out, and the result was a Cronbach’s alpha of 0.778, which is considered adequately reliability.

For the exploratory factor analysis, all of the questions of the questionnaire were included, resulting in a factor solution of 31 items grouped into 8 components with eigenvalues greater than 1, which explains 54.4% of the variance, with commonalities ranging between 0.51 and 0.85. The Kaiser–Meyer–Olkin measure of sampling adequacy was 0.78. Bartlett’s test of sphericity allowed for the rejection of the hypothesis of equality of the matrices (χ^2^(190) = 17591.405; *p* = 0.001). The distribution of the questions is shown in Table 7.

The factor analysis distributed the items into eight components or subcategories, which were as follows: frequencies of mealtimes, physical activity and lifestyle, consumption of high-calorie foods, company and food consumption, consumption of vegetables and fruits, place of food consumption, consumption of alcoholic beverages, and consumption of sugar-sweetened beverages, plain water, and milk. On average, each factor had four items, and the reliability of the components ranged from 0.600 to 0.851, which is considered adequate, and the percentage of variance explained was 53%, which is also considered adequate.

Table 8 presents the results of the factorial analysis of the self-completed questionnaire on eating habits for adolescents in Jalisco and Mexico City. It can be observed that in the case of the application in Mexico City, eight factors resulted, and the factorial loads are in an interval of 0.314 to 0.867, and in the case of Cronbach’s alpha, the minimum was 0.60, and the maximum was 0.85, so it can be said that the scores are slightly higher than those of the questionnaire applied in Jalisco.

## 4. Discussion

The results of the validation of the questionnaire showed an adequate factor structure and internal consistency. Flores and Macedo [16] initially structured the questionnaire into four sections, where the first three assessed eating habits and the last section assessed physical activity habits. However, in this study, when the exploratory factor analysis was conducted, the questionnaire yielded a factor structure of eight sections or factors.

The questionnaire validated in this study identified that most adolescents had partially inadequate eating habits and inadequate physical activity habits. This indicates that these behaviors should be addressed in Mexican adolescents. The results obtained in this study are similar to those reported by Barja-Fernández et al. [26], who reported risky dietary and physical activity patterns in a group of Galician schoolchildren aged 9 to 17 years in relation to Spanish and international recommendations, so the authors urge to continue improving the diet and increase physical sports in this age group. In addition, the results of the study by Alfaro González et al. [27] are partially consistent with this study, as they found that schoolchildren aged 13 to 18 years had inadequate eating practices, low intake of fruits, legumes, fish, and high intake of unhealthy foods, such as sugary drinks, chips, and other ultra-processed foods. However, in contrast with this study, the majority (95.8%) practiced daily physical activity and nearly 70% did so at school. This suggests that in the group of adolescents in this study, the results are discouraging since adolescents consume less recommended food, and there is greater consumption of non-recommended food, and in most adolescents, their physical activity habits are partially inadequate and inadequate. Similarly, ENSANUT-2021 [2] indicates that in Mexico, a high percentage of adolescents have frequent consumption of non-recommended foods, including snacks, sweets, and desserts, in addition to sugar cereals.

According to data reported by ENSANUT-2021 [2], it is worrisome that Mexican adolescents drink more sweetened beverages than plain drinking water, which contributes to the problem of obesity; as for the consumption of recommended foods, about 70% of adolescents do not consume fruits and vegetables, which means an insufficient intake of vitamins, minerals, and fiber, in addition to the fact that only 29.8% have a protein source from egg albumin and less than 25% consume legumes, which are necessary for muscle formation. In our study, 52.4% drink five or more glasses of water daily, while 85% drink at least one glass of sugary drinks every day. These data are similar to those reported by ENSANUT 2021; in terms of vegetables, only 30% consume vegetables between 1 to 6 days and fruits and 31% consume fruits between 1 to 6 days, which also coincides with the data from ENSANUT 2021 [2].

Inadequate eating and physical activity habits lead to being overweight and/or obese. In this study, 28.4% of female adolescents were found to be overweight and 16.1% were found to be obese, and in the case of male adolescents, the prevalence of being overweight and obese was 24.5% in both cases. The ENSANUT survey (2021) [2] reported similar data to this study due to the fact that in Mexico, there are 7.3 million overweight and obese adolescents and this condition has been maintained and has not declined. The highest prevalence of overweight males and females reported in 2020 was 26.9%. Regarding the prevalence of obesity, the highest percentages reported to date were 21.5% for males in 2021 and 17.9% for females in 2020.

Regarding the association between physical activity and healthy eating, our study found that if there is a relationship between physical activity and the consumption of fruits and vegetables, the association found is low, probably because for both physical activity and the consumption of fruits and vegetables, the percentages were also low; however, the importance of associating both behaviors for the benefit of the adolescent population continues to be highlighted.

The results of the economic level of the adolescent population in this study are worrisome, with the majority being at the typical medium level and some at the extremely low level and very extremely low level. In this regard, it is known that the economy has an important influence on the health of adolescents in all aspects of their lives. Income deprivation is associated with the risk of multimorbidity [28] and an increased likelihood of consumption of sugar-sweetened beverages that pervade all of the most vulnerable communities [29]. In addition, recent studies have investigated the role of socioeconomic status in child development, mainly regarding physical and psychological health parameters [30].

Regarding the validation of the questionnaire, the results obtained are similar to those published by Flores and Macedo (2016) [16], who validated the questionnaire in Mexico among adolescents from the State of Jalisco. The similarities were as follows: (a) the factor weights of the work of Flores and Macedo [16] ranged between 0.3 and 0.7 and in this work, they ranged between 0.3 and 0.8., and (b) Cronbach’s alpha ranged between 0.56 and 0.80; this interval was similar to the questionnaire validated in this work.

The differences were that the items were grouped into eight factors, compared to four factors obtained in the validation carried out in Jalisco. On the other hand, in this study, an exploratory factor analysis was performed, but there was no consistency between the test–retest scores due to the number of participants since in the validation in CDMX, the questionnaire was administered to 2710 adolescents, while in the Jalisco study, the sample was of 64 adolescents.

The work carried out with adolescents in Jalisco and Mexico City to validate an instrument on nutrition and physical activity is relevant, and it is necessary to carry out a broader study in other Mexican states in order to have instruments with valid indicators for Mexican students at different educational levels and, based on these, to develop nutritional programs according to the needs of each population group since it is a questionnaire that can assess current eating and physical activity problems, preventive programs can be carried out to avoid more complex situations.

Comparison with other similar works was difficult since in Mexico, there are no studies where instruments for measuring healthy habits and behaviors are developed in the adolescent population, which is why it is important that this type of work continues to be carried out and generates increasingly suitable instruments for this population and that the results of the same studies provide relevant information for work on the prevention of disease in and the care of this population which is at high risk of developing chronic disease problems.

## 5. Study Limitations

The study limitations include:

1. The study was carried out in Mexico City in a specific group of adolescents, which means that the results cannot be generalized to other populations or age groups.
2. Discrepancy between the items of the questionnaire related to eating habits and those related to physical activity.
3. There could be self-report biases in the data obtained through the questionnaire, since the adolescents could have answered inaccurately or misleadingly about their eating habits and physical activity.
4. Imbalance regarding the items of eating habits and physical activity.
5. It should be noted that unfortunately, non-dairy alternatives that have been introduced onto the market were not included in the survey.

## 6. Conclusions

Based on the results obtained in this study, it can be considered that the self-report questionnaire on eating and physical activity habits is reliable since it has adequate internal consistency and can be applied to the adolescent population in Mexico City in future research on healthy lifestyle habits, with a view to encouraging proper nutrition and increased physical activity among adolescents, as part of a healthy lifestyle.

## Figures and Tables

**Table 1:** Self-report questionnaire to evaluate eating and physical activity habits.

The following is a series of questions about your eating and physical activity habits in the last month. You should choose only one answer unless the question indicates otherwise. Remember that there are no right or wrong answers; you just have to answer honestly and select what you normally do.
**Section 1**: How many days a week do you eat vegetables (about 100 g), for example: one cup of sliced cucumber, ½ cup of cooked broccoli, ½ cup of cooked chayote, 2½ cups of lettuce, or one cup of raw carrot? ◦ 0 to 2 days ◦ 3 to 4 days ◦ 5 to 6 days ◦ Daily On the days you do eat vegetables, how many servings (approximately 100 g) do you consume, for example: one cup of sliced cucumber, ½ cup of cooked broccoli, ½ cup of cooked chayote, 2½ cups of lettuce, or one cup of raw carrot? ◦ 1 serving ◦ 2 servings ◦ 3 servings ◦ 4 or more servings How many days a week do you eat fruit (approximately 100 g), for example: two guavas, one tangerine, one medium apple, half a mango, ½ cup of chopped melon, one orange, ½ pear, or ½ banana? ◦ 0 to 2 days ◦ 3 to 4 days ◦ 5 to 6 days ◦ Daily On the days you do eat fruit, how many servings (approximately 100 g) do you consume, for example: two guavas, one tangerine, one medium apple, half a mango, ½ cup of chopped melon, one orange, ½ pear, or ½ banana? ◦ 1 serving ◦ 2 servings ◦ 3 servings ◦ 4 or more servings How many days a week do you drink plain milk, plain yogurt (medium glass of about 240 mL), or 30 g of cheese (which is the size of your whole thumb from tip to base)? ◦ 0 to 2 days ◦ 3 to 4 days ◦ 5 to 6 days ◦ Daily How many glasses of plain drinking water do you drink per day (medium glass of approximately 240 mL)? ◦ 0 to 2 glasses ◦ 3 to 4 glasses ◦ 5 to 6 glasses ◦ 7 or more glasses
**Section 2**: 7. How many days a week do you eat ham, sausage, salami, or chorizo? ◦ 5 or more days ◦ 3 to 4 days ◦ 1 to 2 days ◦ None 8. How many days a week do you eat fast food (hamburgers, pizzas, or tacos) away from home? ◦ 5 or more days ◦ 3 to 4 days ◦ 1 to 2 days ◦ None 9. How many days a week do you eat sweets or chocolates? ◦ 5 or more days ◦ 3 to 4 days ◦ 1 to 2 days ◦ None 10. How many days a week do you eat sweet bread, cookies, or cakes? ◦ 5 or more days ◦ 3 to 4 days ◦ 1 to 2 days ◦ None 11. How many days a week do you eat chips, Doritos nacho cheese tortilla chips, or similar snacks? ◦ 5 or more days ◦ 3 to 4 days ◦ 1 to 2 days ◦ None 12. How many days a week do you drink beer or other alcoholic beverages? ◦ 5 or more days ◦ 3 to 4 days ◦ 1 to 2 days ◦ None 13. On the days you do drink beer or other alcoholic beverages, how much do you consume (medium glass of approximately 240 mL)? ◦ 4 or more glasses ◦ 2 to 3 glasses ◦ 1 glass ◦ None 14. How many days a week do you drink soft drinks, bottled juices, or juice drinks? ◦ 5 or more days ◦ 3 to 4 days ◦ 1 to 2 days ◦ None 15. On the days that you do drink soft drinks, bottled juices, or juice drinks, how many glasses do you consume (medium glass of approximately 240 mL)? ◦ 4 or more glasses ◦ 2 to 3 glasses ◦ 1 glass ◦ None
**Section 3**: Mark how often (days per week) you consume the following mealtimes: 16. Breakfast: ◦ 0 to 1 day ◦ 2 to 3 days ◦ 4 to 5 days ◦ 6 to 7 days 17. Lunch: ◦ 0 to 1 day ◦ 2 to 3 days ◦ 4 to 5 days ◦ 6 to 7 days 18. Dinner: ◦ 0 to 1 day ◦ 2 to 3 days ◦ 4 to 5 days ◦ 6 to 7 days 19. Snacks (small amounts of food consumed between mealtimes): ◦ 0 to 1 day ◦ 2 to 3 days ◦ 4 to 5 days ◦ 6 to 7 days Mark where you usually eat your food (choose only one option per mealtime, whichever is more frequent): 20. Breakfast: ◦ At a street stall or the first stand you find ◦ In a restaurant or establishment ◦ Away from home, the food I bring from home ◦ At home 21. Lunch: ◦ At a street stall or the first stand you find ◦ In a restaurant or establishment ◦ Away from home, the food I bring from home ◦ At home 22. Dinner: ◦ At a street stall or the first stand you find ◦ In a restaurant or establishment ◦ Away from home, the food I bring from home ◦ At home 23. Snack(s) ◦ At a street stall or the first stand you find ◦ In a restaurant or establishment ◦ Away from home, the food I bring from home ◦ At home Mark with whom you usually eat your food (choose only one option per mealtime, whichever is more frequent): 24. Breakfast: ◦ Alone ◦ With acquaintances ◦ With friends ◦ With my family 25. Lunch: ◦ Alone ◦ With acquaintances ◦ With friends ◦ With my family 26. Dinner: ◦ Alone ◦ With acquaintances ◦ With friends ◦ With my family 27. Snack(s): ◦ Alone ◦ With acquaintances ◦ With friends ◦ With my family
**Section 4**: 28. Do you engage in physical activity (from brisk walking to sports)? ◦ Never ◦ Almost never ◦ Frequently ◦ Very often 29. How many hours do you engage in physical activity (from brisk walking to sports) per week? ◦ Less than 2 ◦ From 2 to less than 4 ◦ From 4 to less than 6 ◦ 6 or more 30. Outside of school, how many times a week do you engage in at least 30 min of physical activity (from brisk walking to sports)? ◦ 0 to 2 times ◦ 3 to 4 times ◦ 5 to 6 times ◦ Diary 31. You consider your lifestyle to be: ◦ Very sedentary ◦ Sedentary ◦ Active ◦ Very active

**Table 2:** Criteria for the classification of eating and physical activity habits.

Variable	Category	Designation	Criteria	Score
Eating habits	1	Inadequate	<50% of the maximum possible score	<25.5
	2	Partially inadequate	≥50% and <75% of the maximum possible score	≥25.5 and <38.5
	3	Adequate	≥75% of the maximum possible score	≥38.5
Physical activity habits	1	Inadequate	<50% of the maximum possible score	<6
	2	Partially inadequate	≥50% and <75% of the maximum possible score	≥6 and <9
	3	Adequate	≥75% of the maximum possible score	≥9

**Table 3:** Descriptive results of the sociodemographic and anthropometric variables of the surveyed adolescents.

Variables	Adolescent Males				Adolescent Females				Total			
Baseline age	$\bar{X}$ = 11.7				$\bar{X}$ = 11.7				11 years and 7 months			
Genre	n = 1280 (47.23%)				n = 1430 (52.76%)				n = 2710 (100%)			
NSE A/B *	n = 17 (1.33%)				n = 21 (1.47%)				n = 38 (1.40%)			
NSE C+	n = 262 (20.47%)				n = 276 (19.30%)				n = 538 (19.85%)			
NSE C	n = 502 (39.22%)				n = 565 (39.51%)				n = 1067 (39.37%)			
NSE C−	n = 297 (23.20%)				n = 337 (23.57%)				n = 634 (23.39%)			
NSE D+	n = 120 (9.38%)				n = 137 (9.58%)				n = 257 (9.48%)			
NSE D	n = 79 (6.17%)				n = 91 (6.36%)				n = 170 (6.27%)			
NSE E	n = 3 (0.23%)				n = 3 (0.21%)				n = 6 (0.22%)			
Baseline height	$\bar{X}$ = 157.2				$\bar{X}$ = 153.4				$\bar{X}$ = 155			
Basal weight	$\bar{X}$ = 53.8				$\bar{X}$ = 51.5				$\bar{X}$ = 52.6			
Baseline BMI	$\bar{X}$ = 21.5				$\bar{X}$ = 21.7				$\bar{X}$ = 21.6			
Size-for-age Z-score diagnosis	**Adequate**	**Risk of low height**	**Low height**		**Adequate**	**Risk of low height**	**Low height**		**Adequate**	**Risk of low height**	**Low height**	
	n = 1143 (89.3%)	n = 120 (9.4%)	n = 17 (1.3%)		n = 1153 (80.6%)	n = 253 (17.7%)	n = 24 (1.7%)		n = 2295 (84.7%)	n = 374 (13.8%)	n = 41 (1.5%)	
BMI-for-age Z-score diagnosis	**Underweight**	**Adequate weight**	**Overweight**	**Obesity**	**Underweight**	**Adequate weight**	**Overweight**	**Obesity**	**Underweight**	**Adequate weight**	**Overweight**	**Obesity**
	n = 93 (7.3%)	n = 561 (43.8%)	n = 313 (24.5%)	n = 313 (24.5%)	n = 91 (6.4%)	n = 703 (49.2%)	n = 406 (28.4%)	n = 229 (16.1%)	n = 179 (6.6%)	n = 1238 (45.7%)	n = 705 (26.0%)	n = 588 (21.7%)

* The socioeconomic level variables are presented as: socioeconomic level A/B (high level), socioeconomic level C+ (medium–high level), socioeconomic level C (typical medium level), socioeconomic level C− (emerging medium level), socioeconomic level D+ (typical low level), socioeconomic level D (extremely low level), and socioeconomic level E (very extremely low level) [23].

**Table 4:** Eating habits of adolescent females and adolescent males.

**Adolescent Females**				
**Nutritional Status**	**BMI-for-Age Z-Score Diagnosis**	**Proper Eating Habits**	**Partially Inadequate Eating Habits**	**Inadequate Eating Habits**
Underweight	n = 91 (6.4%)	n = 7 (7.7%)	n = 77 (84.6%)	n = 7 (7.7%)
Normal weight	n = 703 (49.2%)	n = 102 (14.5%)	n = 533 (75.8%)	n = 68 (9.7%)
Overweight	n = 406 (28.4%)	n = 48 (11.8%)	n = 306 (75.4%)	n = 52 (12.8%)
Obesity	n = 230 (16.1%)	n = 20 (8.7%)	n = 184 (80.0%)	n = 26 (11.3%)
**Adolescent Males**				
**Nutritional Status**	**BMI-for-Age Z-Score Diagnosis**	**Proper Eating Habits**	**Partially Inadequate Eating Habits**	**Inadequate Eating Habits**
Underweight	n = 93 (7.3%)	n = 13 (14.0%)	n = 73 (78.5%)	n = 7 (7.5%)
Normal weight	n = 561 (43.8%)	n = 79 (14.1%)	n = 429 (76.5%)	n = 53 (9.4%)
Overweight	n = 313 (24.5%)	n = 51 (16.3%)	n = 238 (76.0%)	n = 24 (7.7%)
Obesity	n = 313 (24.5%)	n = 36 (11.5%)	n = 243 (77.6%)	n = 34 (10.9%)

**Table 5:** Physical activity habits of adolescents.

**Adolescent Females**				
**Nutritional Status**	**BMI-for-Age Z-Score Diagnosis**	**Adequate Physical Activity Habits**	**Partially Inadequate Physical Activity Habits**	**Inadequate Physical Activity Habits**
Underweight	n = 91 (6.4%)	n = 11 (12.1%)	n = 33 (36.3%)	n = 47 (51.6%)
Normal weight	n = 703 (49.2%)	n = 130 (18.5%)	n = 250 (35.6%)	n = 323 (45.9%)
Overweight	n = 406 (28.4%)	n = 65 (16.0%)	n = 153 (37.7%)	n = 188 (46.3%)
Obesity	n = 230 (16.1%)	n = 33 (14.3%)	n = 67 (29.1%)	n = 130 (56.5%)
**Adolescent Males**				
**Nutritional Status**	**BMI-for-Age Z-Score Diagnosis**	**Adequate Physical Activity Habits**	**Partially Inadequate Physical Activity Habits**	**Inadequate Physical Activity Habits**
Underweight	n = 93 (7.3%)	n = 18 (19.4%)	n = 38 (40.9%)	n = 37 (39.8%)
Normal weight	n = 561 (43.8%)	n = 99 (17.6%)	n = 215 (38.3%)	n = 247 (44.0%)
Overweight	n = 313 (24.5%)	n = 71 (22.7%)	n = 102 (32.6%)	n = 140 (44.7%)
Obesity	n = 313 (24.5%)	n = 66 (21.1%)	n = 103 (32.9%)	n = 144 (46.0%)

**Table 6:** Correlations of physical activity and the consumption of fruits and vegetables in adolescents in Mexico City.

	Vegetable Consumption	Fruit Consumption
Physical activity	0.132 **	0.150 **

** The correlation is significant at the 0.05 level (bilateral).

**Table 7:** Results of the factor analysis.

	Mealtime Frequencies	Physical Activity and Lifestyle	Consumption of High-Calorie Foods	Company and Food Consumption	Consumption of Vegetables and Fruits	Place of Food Consumption	Consumption of Alcoholic Beverages	Consumption of Sugar-Sweetened Beverages, Plain Water, and Milk
17. Lunch frequency	0.812							
18. Dinner frequency	0.796							
16. Breakfast frequency	0.754							
19. Snack frequency	0.590							
28. Do you engage in physical activity?		0.786						
30. How many times a week do you engage in at least 30 min of physical activity?		0.779						
29. How many hours do you engage in physical activity?		0.731						
31. Lifestyle		0.648						
11. How many days a week do you eat chips, Doritos nacho cheese tortilla chips, or similar snacks?			0.726					
9. How many days a week do you eat sweets or chocolates?			0.720					
10. How many days a week do you eat sweet bread, cookies, or cakes?			0.675					
14. How many days a week do you drink sugary beverages?			0.636					
8. How many days a week do you eat fast food?			0.431					
7. How many days a week do you eat ham, sausage, salami, or chorizo?			0.370					
26. With whom do you eat dinner?				0.738				
27. With whom do you eat your snacks?				0.719				
24. With whom do you eat breakfast?				0.704				
25. With whom do you eat lunch?				0.636				
2. On the days you do eat vegetables, how many servings do you eat?					0.697			
4. On the days you do eat fruit, how many servings do you eat?					0.694			
3. How many days a week do you eat fruit?					0.665			
1. How many days a week do you eat vegetables?					0.631			
22. Where do you eat dinner?						0.697		
23. Where do you eat snacks?						0.689		
21. Where do you eat lunch?						0.664		
20. Where do you eat breakfast?						0.619		
12. How many days a week do you drink beer or other alcoholic beverages?							0.867	
13. On the days you do drink beer or other alcoholic beverages, how much do you consume?							0.862	
15. On the days you do drink soft drinks, bottled juices, or juice drinks, how many glasses do you consume?								−0.768
6. How many glasses of plain drinking water do you drink per day?								0.511
5. How many days a week do you drink plain milk, plain yogurt, or cheese?								0.314
Total = Cronbach’s alpha of 0.778								
Number of items	4	4	6	4	4	4	2	3
% of variance explained	7.69%	15.2%	22.6%	29.5%	36.2%	42.8%	49.2%	53%
Cronbach’s alpha	0.756	0.735	0.679	0.679	0.637	0.663	0.851	0.600

**Table 8:** Factorial structure and internal consistency of the self-completed questionnaires on eating habits for adolescents from Jalisco and Mexico City.

	Self-Completed Questionnaire on Eating Habits for Adolescents in Jalisco	Factor Structure	
*Section*	*Questionnaire factors*	*Factorial Loads* *(min–max)*	*Cronbach’s alpha*
1	Recommended food intake	0.41–0.64	0.56
2	Recommended food intake	0.40–0.77	0.73
3	Eating behaviors	0.32–0.75	0.80
1–3	Total, eating habits	----	0.71
4	Physical activity	0.56–0.78	0.76
	Self-completed questionnaire on eating habits for adolescents in Mexico City	Factor structure	
*Section*	*Questionnaire factors*	*Factorial Loads* *(min–max)*	*Cronbach’s alpha*
1	Mealtime frequencies	0.590–0.812	0.75
2	Physical activity and lifestyles	0.648- 0.786	0.73
3	Consumption of high-calorie foods	0.370- 0.726	0.67
4	Company and food consumption	0.636–0.738	0.67
5	Vegetable and fruit consumption	0.631–0.697	0.63
6	Place of consumption of food	0.619–0.697	0.66
7	Consumption of alcoholic beverages	0.862–0.867	0.85
8	Consumption of sugary drinks, plain water, and milk	0.314–-0.768	0.60

## Data Availability

Data sharing not applicable.
